# Antiadipogenic Effects of Mixtures of *Cornus officinalis* and *Ribes fasciculatum* Extracts on 3T3-L1 Preadipocytes and High-Fat Diet-Induced Mice

**DOI:** 10.3390/molecules25102350

**Published:** 2020-05-18

**Authors:** Eunkuk Park, Chang Gun Lee, Hyesoo Jeong, Subin Yeo, Ji Ae Kim, Seon-Yong Jeong

**Affiliations:** 1 Department of Medical Genetics, Ajou University School of Medicine, Suwon 16499, Korea; jude0815@hotmail.com (E.P.); dangsunsang@naver.com (C.G.L.); 2 Department of Biomedical Sciences, Ajou University Graduate School of Medicine, Suwon 16499, Korea; 3 Nine B Company, Daejeon 34121, Korea; jhyesoo921@gmail.com (H.J.); snsnans@naver.com (S.Y.); ji.ae.kim@daum.net (J.A.K.)

**Keywords:** obesity, *Cornusofficinalis*, *Ribesfasciculatum*, high-fat diet, natural product

## Abstract

Medicinal plants have been used worldwide as primary alternative healthcare supplements. *Cornus officinalis* (CO) and *Ribes fasciculatum* (RF) are traditional medicinal plants applied in East Asia to treat human diseases such as hepatitis, osteoporosis, oxidative stress and allergy. The aim of this study was to examine the anti-obesity effect of CO and RF on preadipocyte 3T3-L1 cells in vitro and high-fat diet (HFD)-induced obesity mice in vivo. Combination treatment of CO and RF in differentiated 3T3-L1 cells inhibited adipocyte differentiation through downregulation of adipogenesis-associated genes such as CCAAT/enhancer-binding protein alpha (*Cebpa*), fatty acid binding protein 4 (*Fabp4*), peroxisome proliferator-activated receptor gamma *(Pparg*) and sterol regulatory element binding protein (*Srebp1*). In vivo animal models showed that a mixture of CO and RF inhibited HFD-induced weight gain, resulting in decreased abdominal visceral fat tissues and fatty hepatocyte deposition. In addition, CO+RF treatment decreased HFD-induced adipogenesis-associated genes in abdominal white fat tissue. These results suggest that administration of a CO and RF mixture prevented adipocyte differentiation and lipid accumulation in preadipocyte cells and HFD-induced body weight in obesity mice. Therefore, combined therapy of CO and RF may be a protective therapeutic agent against obesity.

## 1. Introduction

Obesity is one of the greatest health issues in modern society. Obesity is a state of energy imbalance caused by factors such as excessive uptake of energy or insufficient consumption, hormone changes, or genetic, mental and socioeconomic factors [1,2]. Imbalanced energy results in serious health problems including hypertension, diabetes mellitus and atherosclerosis [3,4]. A well-characterized feature of obesity is accumulation of fat in the body [5]. Preadipocytes derived from mesenchymal stem cells differentiate into adipocytes via adipogenesis gene-inducing transcription factors such as peroxisome proliferator-activated receptor-gamma (*Pparg*) and CCAAT/enhancer-binding family of proteins (C/EBPs) [6]. Differentiated adipocytes stimulate fat accumulation into semiliquids, triglycerides and cholesteryl esters [7]. Therefore, excessive lipid-accumulation observed in obesity causes increased adipocyte and steatosis [8,9].

Although several treatments for obesity are suggested, including diet change and physical activity, patients with severe obesity are recommended for active treatment such as drugs or surgical therapy [10,11]. Several anti-obesity medicinal therapies are used for weight loss but have adverse effects, such as headache, nausea, dizziness and fatigue [12,13]. Natural products have emerged as an alternative modern therapy for diseases such as cancer, hypertrophy, diabetes mellitus, burning sensation and cardiovascular disease because they are culturally appropriate sources of primary health care with few side effects [14,15,16]. Studies report that combined natural products promote advantages of synergistic effects on prevention of side effects compared to single treatments [17,18,19,20]. For instance, combination therapy of *Cornus officinalis* and *Rehmannia radix* has synergistic effects on the kidneys in diabetes-induced renal injury [21]. In addition, an herbal mixture of five plant extracts has complex effects on atopic dermatitis [22]. 

*Cornus officinalis* (CO) has been widely used as a commercialized raw material for tea and health supplements as an herbal medicine [23]. Many studies demonstrate protective effects of CO on hepatitis, osteoporosis, oxidative stress and obesity [24,25,26]. *Ribes fasciculatum* (RF) is also a common alternative traditional medication against inflammatory conditions such as allergy, aging and autoimmunity in Asia [27,28,29]. Despite the beneficial effects of these natural plants, combination CO and RF extracts for weight loss have not been reported.

We examined anti-obesity effects of CO and RF in preadipocyte 3T3-L1 cells in vitro and high-fat diet (HFD)-induced obesity mice in vivo. We found that combined CO and RF reduced adipogenesis of preadipocyte and HFD-induced obesity in mice.

## 2. Results

### 2.1. Effects of CO and RF on Adipogenesis in Vitro

**Figure 1:**
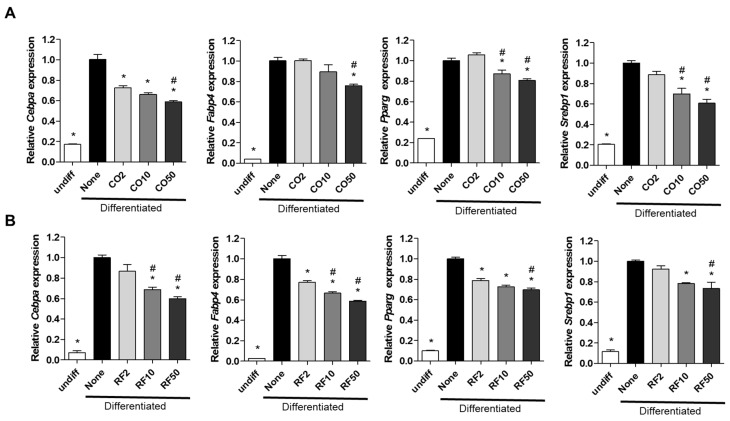
Anti-adipogenic effects of *Cornus officinalis* (CO) and *Ribes fasciculatum* (RF) on mRNA expression of adipogenic markers in 3T3-L1 cells. Cells were induced by 3-isobutyl-1-methylxanthine, dexamethasone and insulin multiple daily injections and co-incubated with CO (A) and RF (B) at 2, 10, or 50 μg/mL during lipid accumulation. mRNA of adipogenesis-associated genes was analyzed by qRT-PCR. Relative mRNA of *Cebpa*, *Fabp4*, *Pparg* and *Srebp1* was normalized by *Gapdh*. * *p* < 0.05 vs. none, # *p* < 0.05 vs. CO2 or RF2 (Tukey’s honest significant difference post hoc test, analysis of variance). Abbreviations: Undiff—undifferentiated; None—non-treated.

We examined the anti-obesity effects of CO and RF on 3T3-L1 preadipocyte cells. Differentiation of preadipocytes was induced with 3-isobutyl-1-methylxanthine, dexamethasone and insulin multiple daily injections (MDI) as previously described [30] with cotreatment with either CO or RF extracts at four concentrations (0, 2, 10 and 50 μg/mL). Adipocyte differentiation was measured as mRNA expression of adipogenic markers such as CCAAT/enhancer-binding protein alpha (*Cebpa*), fatty acid binding protein 4 (*Fabp4*), peroxisome proliferator-activated receptor gamma *(Pparg*) and sterol regulatory element binding protein (*Srebp1*). CO and RF extracts did not affect the viability of 3T3-L1 cells (Figure S1). Both CO and RF extracts decreased adipogenesis-inducing genes (Figure 1A,B), suggesting inhibition of adipocyte differentiation. 

### 2.2. Effects of CO+RF Extract Mixture on Adipogenesis in 3T3-L1 Cells

**Figure 2:**
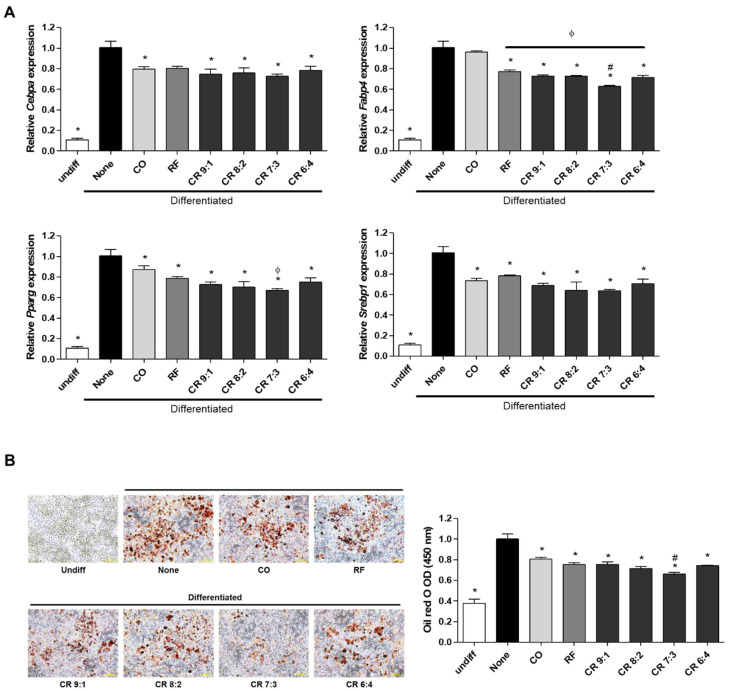
Anti-adipogenic effects of combined treatment with CO and RF in 3T3-L1 cells. After induction of adipogenesis, cells were treated with combined CO and RF at indicated ratios (9:1, 8:2, 7:3 and 6:4) at two concentrations (10 and 50 μg/mL). (**A**) The mRNA levels of adipogenesis-associated genes (*Cebpa*, *Fabp4*, *Pparg* and *Srebp1*) were analyzed by qRT-PCR. Relative mRNA was normalized to mouse *Gapdh*. (**B**) Lipid-accumulation of adipocytes assessed by oil red O staining and quantification of lipid accumulation, based on optical density (OD) value for destained oil red O. Abbreviations: Undiff, undifferentiated; None, non-treated. * *p* < 0.05 vs. none, # *p* < 0.05 vs. RF, φ *p* < 0.05 vs. CO (Tukey’s honest significant difference post hoc test, analysis of variance).

To further determine the synergistic effect of CO and RF, we evaluated the anti-obesity effects of CO and RF on MDI-induced preadipocyte differentiation. Cells were treated with CO and RF mixture at ratios of 9:1, 8:2, 7:3 and 6:4 at 10 and 50 μg/mL, and mRNA of adipogenic markers (*Cebpa*, *Fabp4*, *Pparg* and *Srebp1*) was assessed by ΔCt (Figure S2). Significant decreases in adipogenic markers were observed for all ratios of CO and RF mixture (9:1, 8:2, 7:3 and 6:4) and both concentrations (10 and 50 μg/mL). Since differences between 10 and 50 μg/mL were not constantly significant (Figure S2), for efficiency of the natural products, we selected lower concentrations of the CO and RF mixture (10 μg/mL) for subsequent experiments, and then the mixture extract was compared with a single treatment of CO and RF. All CO and RF combinations and single treatment with CO and RF reduced mRNA of adipogenic markers. A mixture of CO and RF at 10 μg/mL had synergistic inhibitory effects on adipogenic-inducing genes compared to single extracts of CO and RF. The greatest effect on mRNA from *Fabp4* and *Pparg* occurred at a 7:3 ratio of 10 μg/mL CO and RF extract (Figure 2A). We tested lipid accumulation of differentiated adipocytes using oil red O staining. Treatment of differentiated 3T3-L1 cells with CO and RF extracts reduced positive-oil red O cells and decreased lipid accumulation optical density (OD) value, compared to MDI alone (Figure 2B), resulting in attenuated lipid droplet accumulation. These results suggest that adipocyte differentiation and lipid accumulation in MDI-induced preadipocyte cells were inhibited by CO+RF mixture at a 7:3 ratio of 10 μg/mL.

### 2.3. Anti-Obesity Effects of CO and RF in HFD-Induced Murine Obesity Model

**Figure 3:**
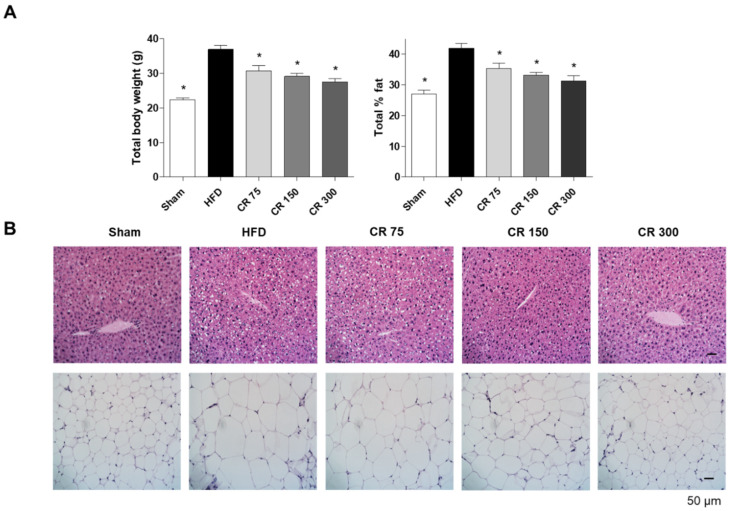
Anti-obesity effects of combined CO and RF in high-fat diet (HFD)-induced obesity murine model. Mice were given normal diet (Sham) or 60% fat diet (HFD) with co-administration of CO and RF at indicated concentrations (75, 150 and 300 mg/kg). (**A**) Total body weight and total % fat was analyzed using an electronic scale and a PIXI-mus small animal densitometer, respectively. (**B**) Formalin-fixed paraffin-embedded tissues from mouse liver and adipose tissue were stained using hematoxylin and eosin (H&E). Representative images were visualized under a light microscope; scale bar, 50 μm. * *p* < 0.05 vs. HFD.

Based on the in vitro results, we determined the anti-obesity effects of CO and RF mixture at a 7:3 ratio on HFD-induced-obesity mice. Four-week-old male C57BL/6J mice were provided with a 60% fat diet to induce obesity and divided into five groups: (1) Control (normal diet); (2) HFD (60% high-fat diet); (3) CO+RF 75 (60% high-fat diet with 75 mg/kg CO and RF mixture); (4) CO+RF 150 (60% high-fat diet with 150 mg/kg CO and RF mixture); and (5) CO+RF 300 (60% high-fat diet with 300 mg/kg CO and RF mixture). Each concentration of CO and RF extracts was mixed with 60% fat diet and normal diet, and administration was daily for 12 weeks. Food intake did not differ among control, HFD and CO+RF (75, 150 and 300) groups (data not shown). HFD-induced obesity mice were characterized by increased body weight and total % fat [31], concurrent with increased hepatic steatosis [32,33]. At the end of experiments, we examined body weight and total % fat, and histological images of abdominal fat and liver tissues were visualized by hematoxylin and eosin (H&E) staining. HFD-induced obesity mice showed elevated total body weight and total % fat (Figure 3A) and increased hepatic steatosis and enlarged adipose cells (Figure 3B). Co-administration of CO and RF for 12 weeks inhibited HFD-induced body weight gain and total % fat and decreased fatty deposition in hepatocytes and adipose cell diameter in adipocytes, compared to HFD only (Figure 3A,B). These results indicate that co-administration of CO and RF ameliorated HFD-induced obesity in mice.

**Figure 4:**
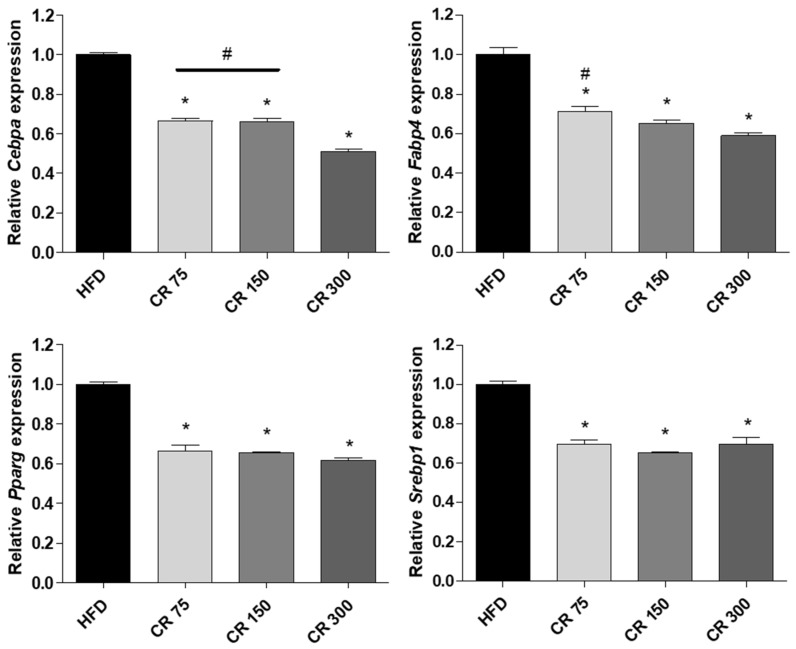
Anti-adipogenic effects of combined CO and RF in murine obesity model. White fat tissue was isolated from abdominal fat, and mRNA of adipogenesis-associated genes in tissue was analyzed by qRT-PCR. Relative mRNA expression levels of *Cebpa*, *Fabp4*, *Pparg* and *Srebp1* were normalized to that of *Gapdh*. * *p* < 0.05 vs. HFD, # *p* < 0.05 vs. CR 300 (Tukey’s honest significant difference post hoc test, analysis of variance).

To confirm the anti-obesity effect of combined CO and RF in HFD-induced mice, we examined the mRNA of adipogenesis-associated genes *Cebpa*, *Fabp4*, *Pparg* and *Srebp1* in white fat tissue. Tissues were collected from mouse abdominal fat, and adipogenesis-associated genes were assessed by qRT-PCR. The results were similar to those of in vitro studies showing that combined administration of CO and RF in HFD-induced obese mice promoted the downregulation of adipogenesis-associated genes *Cebpa*, *Fabp4*, *Pparg* and *Srebp1* compared to those in HFD-induced-obesity mice (Figure 4). These results support our hypothesis that combined CO and RF extracts inhibit HFD-induced obesity by downregulation of adipogenesis-related genes.

### 2.4. HPLC Profile of CO and RF Extracts

**Figure 5:**
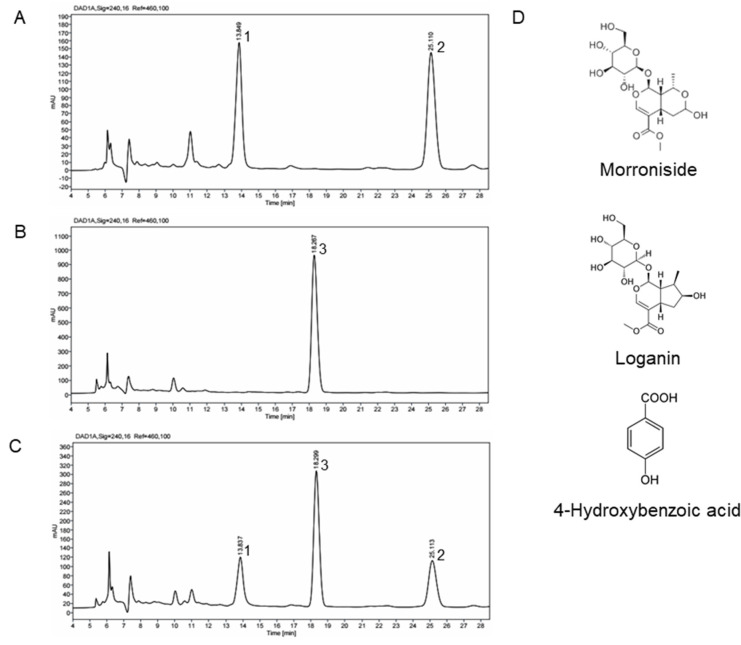
HPLC-diode array detector (DAD) chromatograms showing the presence of morroniside (1), loganin (2) and 4-hydroxybenzoic acid (3) in extracts of CO (**A**), RF (**B**) and a combination of CO and RF (7:3) (**C**). Chromatogram peaks were identified using reference chemicals. Chemical structures (**D**) of loganin, morroniside and 4-hydroxybenzoic acid in extracts of CO, RF and a combination of CO and RF (7:3).

The HPLC profiles of extracts of CO, RF and a combination of CO and RF (7:3) were investigated to obtain overall qualitative information (Figure 5A–C). The identification of the major compound in CO and RF extracts was by HPLC-diode array detector (DAD). Evaluation of peaks corresponding to each compound was by commercially available reference chemicals. We identified morroniside and loganin from CO extracts (Figure 5A), which are the major iridoid components found in extracts of CO. The two compounds were previously shown to have pharmacological effects on osteoporosis and neuroprotection [34,35]. The major compound isolated from RF extract was determined using HPLC (Figure 5B). Peaks corresponding to 4-hydroxybenzoic acid were confirmed by comparison to retention times of standard compounds. In a previous study, 4-hydroxybenzoic identified in ethanol extracts of RF exhibited neuroprotective and anti-inflammatory effects [36,37]. Morroniside, 4-hydroxybenzoic acids and loganin in a combination of CO and RF (7:3) were eluted at retention times of 13.6, 24.0 and 18.0 min, respectively, under our analytical conditions (Figure 5C). Chemical structures of the major components in CO and RF extracts with chemical names are shown in Figure 5D.

## 3. Discussion

Obesity is strongly associated with metabolic diseases such as diabetes, hypertension, cardiovascular disease and cancers [38]. Various approaches to obesity treatment are required for weight loss and prevention of obesity-related diseases. In this study, we examined the anti-obesity effects of CO and RF on in vitro and in vivo models.

Adipogenesis is induced by the increased expression of adipogenesis-inducing genes such as *Cebpa*, *Fabp4*, *Pparg* and *Srebp1.* Adipocyte differentiation is initiated by induction of *Pparg* [39]. A study reported that *Pparg* knockout resulted in decreased adipocyte size and liver steatosis [40]. A main target gene of *Pparg* is the lipid transporter *Fabp4*, expressed primarily in adipocytes as a fatty acid chaperone [41]. In addition, *Cebpa* is modulated by *Pparg* associated with adipocyte differentiation and lipid accumulation of adipocytes [42,43,44,45]. *Srebp1* transcription factors are major regulators of lipid homeostasis that control endogenous cholesterol, triacylglycerol, fatty acid and phospholipid synthesis [46]. These adipogenic-related genes stimulate adipocyte differentiation of 3T3-L1 cells and differentiated adipocytes were apparently by lipid-rich accumulation, which is histologically stained by oil red O [47]. In previous studies, a CO-containing formulation inhibited the expression of *Pparg* and *Cebpa* [48] and a mixture of CO and RF extracts reduced ovariectomized (OVX)-induced weight gain in mice [49]. Our results show that CO and RF extracts significantly suppressed the expression of *Pparg* and *Cebpa,* which may, in turn, decrease the expression of *Fabp4,* because *Fabp4* is a target gene of *Pparg* and *Cebpa.* These findings suggest that treatment with combined CO and RF effectively decreased the adipocyte differentiation of 3T3-L1 preadipocytes and reduced oil red O-positive cells by inhibiting the expression of adipogenesis-inducing genes.

HFD-induced hyperplasia and hypertrophy of white adipose tissue are crucial in development of obesity murine models. In addition, excessive intake of fat in HFD-induced obesity mice triggers hepatic steatosis, correlated with adipogenesis-associated genes [50,51]. However, studies showed that HFD-administered *Pparg* or C/EBP-deficient mice presented decreased adipocyte hypertrophy, resulting in loss of body weight [52,53]. In our study, administration of combined CO and RF in obese mice inhibited HFD-induced weight gain with decreased liver steatosis and adipocyte size by reducing adipogenesis-associated genes. As only female mice were included in our experiment, it is unclear whether the anti-obesity effect of combined CO and RF extracts is gender-specific.

In summary, combined treatment of CO and RF at a 7:3 ratio showed an optimal inhibitory effect on adipogenesis-associated genes (*Cebpa*, *Fabp4*, *Pparg* and *Srebp1*) in 3T3-L1 preadipocytes, compared to single treatment. In addition, oil red O-positive staining cells were decreased, indicating reduced lipid accumulation and adipocyte differentiation. In an HFD-induced mouse obesity model, administration of combined CO and RF resulted in significant decrease in body weight, total % fat, liver steatosis and size of white adipose tissue.

## 4. Materials and Methods

### 4.1. Preparation of CO and RF Extracts

CO material was obtained from Icheon and Yangpyeon (Gyeonggi-do, South Korea). RF material was obtained from Goesan (Chungcheongbuk-do, South Korea). Air-dried CO (100 g) and RF (50 g) were extracted with ethanol solution in water bath. The extracts were filtered with qualitative low ash filter paper (CHMLAB, Terrassa, Barcelona, Spain) to remove debris. The filtrate was collected and concentrated in a rotating evaporator and lyophilized under reduced pressure. The dried extract was stored in glass bottles at −20 °C before the experiments. The ethanol extract of CO or RF powder was resuspended with distilled water and filtered through a BioFil™ 0.45 μm PVDF syringe filter (Microlab Scientific, Mongkok, Honkong).

### 4.2. HPLC-DAD Analysis of CO and RF Extracts

Ethanol extracts of CO or RF powder (1 g) were dissolved in 100 mL 50% methanol and filtered through a 0.45-μm PTFE syringe filter. Filtrate was used for high performance liquid chromatography (HPLC). The Agilent HPLC system (Agilent Technologies, Palo Alto, CA, USA) consisted of an Agilent 1290 quaternary pump, Agilent 1290 vial sampler and a Hypersil GOLD™ C_18_ reverse-phase HPLC column (4.6 × 250 mm, 5 μm, Thermo Scientific, San Jose, CA, USA). The mobile phase consisted of 0.1% acetic acid in solvent A (water: acetonitrile: methanol, 85:10:5, *v/v/v*) and solvent B (acetonitrile). The gradient elution was: 0–20 min 0% B, 20–25 min 80% B, 25–30 min 80% B, 30–30.1 min 0% B and 30.1–38 min 100% B. The flow rate was 0.5 mL/min and column temperature maintained at 30 °C. Sample injection volume was 10 mL. Diode array detector (DAD) acquisitions were performed in the 190–400 nm range, and chromatograms were integrated at 240 nm.

### 4.3. Cell Culture and Adipocyte Differentiation

The mouse 3T3-L1 cell line was from the Korean Cell Line Bank (KCLB, Seoul, Korea). Cells were maintained with Dulbecco’s modified Eagle’s medium (DMEM, Welgene, Gyeongsan, Korea) containing 10% fetal bovine serum (FBS, Gibco, Grand Island, NY, USA), 100 U/mL penicillin and 100 μg/mL streptomycin (Gibco, Grand Island, NY, USA). All cultured cells were incubated in a humidified 37 ℃ atmosphere containing 5% CO_2_. For adipocyte differentiation, pre-adipocyte 3T3-L1 cells were incubated with DMEM containing 0.5 mM 3-isobutyl-1-methylxanthine, 1 mM dexamethasone and 1 μg/mL insulin. After 3 days, cells were incubated with 1 μg/mL insulin for 5 days of lipid accumulation.

### 4.4. Cell Viability

The 3T3-L1 cells (1 × 10^4^ cells per well) were incubated in 96-well plates overnight. To confirm the effect of compounds on cytotoxicity on 3T3-L1 pre-adipocytes, cells were incubated with CO and RF (2, 10, or 50 μg/mL) for 24 h. Cell viability was determined by EZ-Cytox Cell Viability Assay kits (Daeil; Seoul, Korea) under the manufacturer’s instructions. Absorbance was measured at wavelength 450 nm using a microplate reader (Bio-Rad, Hercules, CA, USA).

### 4.5. Quantitative Reverse-Transcription Polymerase Chain Reaction 

Total RNA was extracted using TRIzol (Invitrogen, Carlsbad, CA, USA) reagent and complementary DNA (cDNA) was synthesized using cDNA Synthesis kits (Thermo Fisher Scientific, Waltham, MA, USA), following the manufacturer’s instructions. CDNA was mixed with TB Green TM Premix Ex Taq^TM^ (TaKaRa, Shiga, Japan), and quantitative reverse-transcription polymerase chain reaction (qRT-PCR) was performed using a CFX Connet^TM^ Real-Time System (BIO-RAD, Hercules, CA, USA). Primer sequences for the adipogenesis-related genes were: 5’-GCG GGA ACG CAA CAA CAT C-3’ and 5’-GTC ACT GGT CAA CTC CAG CAC-3’ for mouse *Cebpa*, 5’-AAG GTG AAG AGC ATC ATA ACC CT-3’ and 5’-TCA CGC CTT TCA TAA CAC ATT CC-3’ for mouse *Fabp4*, 5’-GGA AGA CCA CTC GCA TTC CTT-3’ and 5’-GTA ATC AGC AAC CAT TGG GTC A-3’ for mouse *Pparg*, 5’-AAG ATG TAC CCG TCC GTG TC-3’ and 5’-TGA AGG CAG GCT CGA GTA AC-3’ for mouse *Srebp1*. Relative gene expression levels were quantified and normalized to mouse *Gapdh*. Mouse *Gapdh* primers were: 5’-AGG TCG GTG TGA ACG GAT TTG-3’ and 5’-TGT AGA CCA TGT AGT TGA GGT CA-3’. Results were presented as 2^−ΔΔCt^ (ΔΔCt=ΔCt_treatment_ – Ct _control_), and fold-change was determined by comparison to the non-treated adipocyte-differentiated group (none group).

### 4.6. Oil Red O Staining of Adipocytes

After adipogenesis, lipid-accumulation was measured by oil red O staining. Differentiated cells were fixed by 4% paraformaldehyde (PFA) for 15 min and washed with phosphate-buffered saline (PBS) three times. Then, cells were stained with oil red O dye for 2 h. Stained lipid droplets of adipocytes were observed under a microscope. To quantify lipid accumulation, oil red O-stained cells were extracted using isopropanol and incubated for 10 min. The absorbance of the extracted dye was measured at 450 nm (BIO-RAD; Hercules, CA, USA).

### 4.7. In Vivo Experiments in an Obesity Murine Model

Four-week-old female C57BL/6J mice were from DBL Co., Ltd (Chungbuk, Korea). Mice were maintained on high-fat diet (3.0–5.0 g/day) (DooYeol Biotech, Seoul, Korea) and tap water (15 mL/day) for 12 weeks. All mice were housed individually in transparent plastic cages under controlled temperature (23 ± 2 °C), humidity (55 ± 5%) and illumination (12 h light/dark cycle). Mixtures of CO and RF at different concentrations were administrated by oral gavage (2, 10 and 50 mg/kg/day, *n* = 5 in each group). After the experiment, body weight was calculated using an electronic scale, and total fat was measured using a PIXI-mus small animal densitometer with on-board PIXI-mus software (GE Lunar, Little Chalfont, UK), adjusted to body weight. Experiments were approved by the Institutional Animal Care and Use Committee of Ajou University School of Medicine (2016-0062). All experiments were conducted according to the institutional guidelines of the committee.

### 4.8. Preparation of Tissue Samples and Histology

Mouse abdominal fat and liver tissues were excised and fixed in 4% PFA in 0.1 M phosphate buffer at pH 7.4, and samples were embedded in paraffin. Transverse sections (3 μM) of tissue were obtained. Sections were deparaffinized with xylene, rehydrated with ethanol and stained with H&E to evaluate histological changes in the fat and liver tissues.

### 4.9. Statistical Analysis

Statistical analyses were performed with statistical software package SPSS 11.0 for Windows (SPSS Inc., Chicago, IL, USA). Statistical significance of differences was assessed by the Student’s *t*-test. In addition, multiple groups were analyzed using one-way analysis of variance (ANOVA) with Tukey’s HSD post-hoc test. A value of *p* < 0.05 was considered to be statistically significant (* *p* < 0.05). Averages and error bars show standard error of the mean (SEM).

## 5. Conclusion 

This study examined an anti-obesity effect of combined CO and RF through in vitro and in vivo experimental models. We suggest that combined CO and RF may be a potential agent for anti-obesity medications.

## Supplementary Materials

The following are available online at https://www.mdpi.com/1420-3049/25/10/2350/s1: Figure S1. Effect of *Cornus officinalis* (CO) or *Ribes fasciculatum* (RF) on cell viability in 3T3-L1 cells. Cells were treated with CO or RF for 48 h, and cell viability was assessed by WST assay. Figure S2: Anti-adipogenic effect of combined treatment with CO and RF of preadipocytes. The 3T3-L1 cell line was treated with several CO and RF mixtures (10 and 50 μg/mL). Adipogenesis-associated genes (*Cebpa*, *Fabp4*, *Pparg* and *Srebp1)* were evaluated by qRT-PCR. Relative mRNA was normalized to that of mouse *Gapdh*. * *p* < 0.05 vs. none, # *p* < 0.05 vs. CR10 (Tukey’s honest significant difference post hoc test, analysis of variance). Abbreviations: Undiff, undifferentiated; None, non-treated.
