# Supplementary material for: Antiadipogenic Effects of Mixtures of Cornus officinalis and Ribes fasciculatum Extracts on 3T3-L1 Preadipocytes and High-Fat Diet-Induced Mice
Source: Molecules. 2020 May 18;25(10):2350. doi: 10.3390/molecules25102350 (PMC7287608; doi:10.3390/molecules25102350)
Supplement: Supplementary file 1 [file molecules-25-02350-s001.pdf]

Supplementary Materials

Antiadipogenic Effects of Mixtures of *Cornus officinalis* and *Ribes fasciculatum* Extracts on 3T3-L1 Preadipocytes and High-Fat Diet-Induced Mice

Eunkuk Park [1,2,†], Chang Gun Lee [1,2], Hyesoo Jeong [3], Subin Yeo [3], Ji Ae Kim [3] and Seon-Yong Jeong [1,2,*]

[1] Department of Medical Genetics, Ajou University School of Medicine, Suwon 16499, Republic of Korea; jude0815@hotmail.com (E.P.); dangsunsang@naver.com (C.G.L.)
[2] Department of Biomedical Sciences, Ajou University Graduate School of Medicine, Suwon 16499, Republic of Korea
[3] Nine B Company, Daejeon 34121, Republic of Korea; jhyesoo921@gmail.com (H.J.), snsnans@naver.com (S.Y.), ji.ae.kim@daum.net (J.A.K.)
* Correspondence: jeongsy@ajou.ac.kr; Tel.: +82-31-219-4520; Fax: +82-31-219-4521

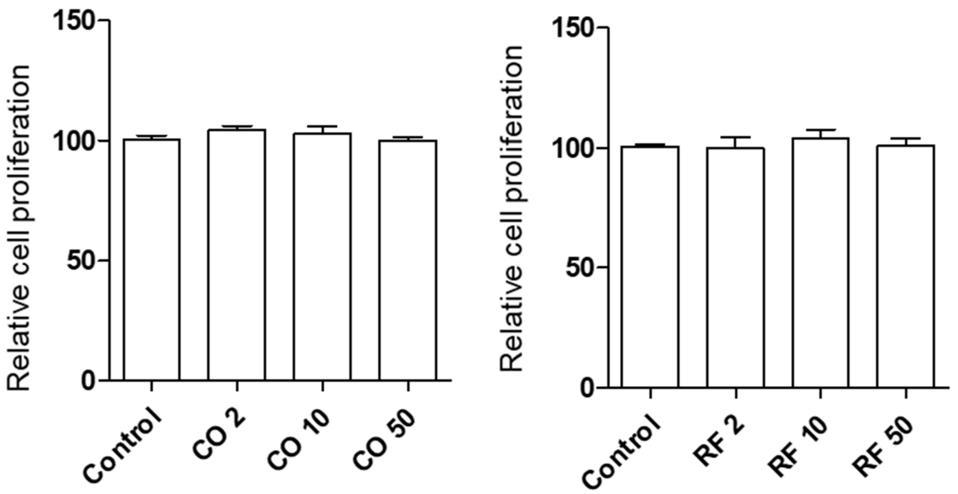

Figure S1. Effect of *Cornus officinalis* (CO) or *Ribes fasciculatum* (RF) on cell viability in 3T3-L1 cells. Cells were treated with CO or RF for 48 h and cell viability was assessed by WST assay.

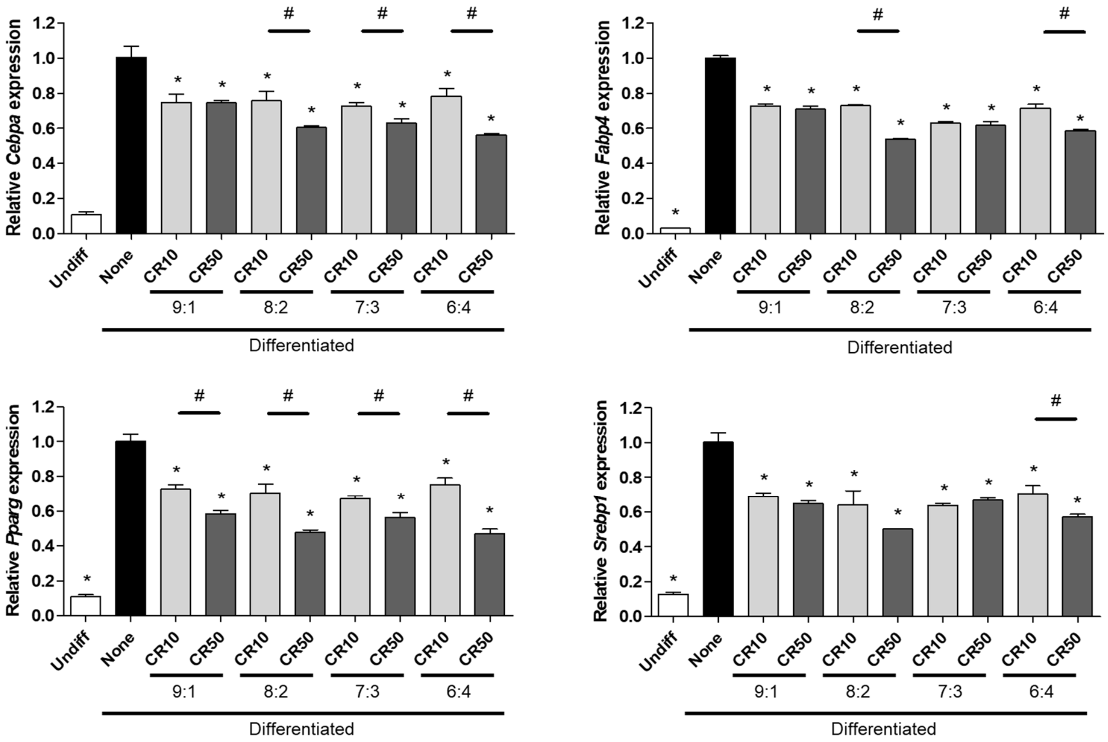

Figure S2. Anti-adipogenic effect of combined treatment with CO and RF in preadipocyte. 3T3-L1 cells were treated with several conditions of CO and RF mixture (10 and 50 μg/ml). Adipogenesis-associated genes (*Cebpa*, *Fabp4*, *Pparg* and *Srebp1)* were evaluated by qRT-PCR. The relative mRNA expressions were normalized by mouse *Gapdh*. * $p < 0.05$ vs. none, # $p < 0.05$ vs. CR10 (Tukey's honest significant difference post hoc test, analysis of variance). Abbreviations: Undiff, undifferentiated; None, non-treated.
